# Potential Use of ChatGPT for Patient Information in Periodontology: A Descriptive Pilot Study

**DOI:** 10.7759/cureus.48518

**Published:** 2023-11-08

**Authors:** Osman Babayiğit, Zeynep Tastan Eroglu, Dilek Ozkan Sen, Fatma Ucan Yarkac

**Affiliations:** 1 Department of Periodontology, Necmettin Erbakan University, Faculty of Dentistry, Konya, TUR

**Keywords:** chatgpt, chat generative pre-trained transformer, large language models (llms), patient information, oral medicine and periodontology, dental care, artificial intelligence in dentistry

## Abstract

Objectives

The aim of this study is to evaluate the accuracy and completeness of the answers given by Chat Generative Pre-trained Transformer (ChatGPT) (OpenAI OpCo, LLC, San Francisco, CA), to the most frequently asked questions on different topics in the field of periodontology.

Methods

The 10 most frequently asked questions by patients about seven different topics (periodontal diseases, peri-implant diseases, tooth sensitivity, gingival recessions, halitosis, dental implants, and periodontal surgery) in periodontology were created by ChatGPT. To obtain responses, a set of 70 questions was submitted to ChatGPT, with an allocation of 10 questions per subject. The responses that were documented were assessed using two distinct Likert scales by professionals specializing in the subject of periodontology. The accuracy of the responses was rated on a Likert scale ranging from one to six, while the completeness of the responses was rated on a scale ranging from one to three.

Results

The median accuracy score for all responses was six, while the completeness score was two. The mean scores for accuracy and completeness were 5.50 ± 0.23 and 2.34 ± 0.24, respectively. It was observed that ChatGPT's responses to the most frequently asked questions by patients for information purposes in periodontology were at least "nearly completely correct" in terms of accuracy and "adequate" in terms of completeness. There was a statistically significant difference between subjects in terms of accuracy and completeness (P<0.05). The highest and lowest accuracy scores were peri-implant diseases and gingival recession, respectively, while the highest and lowest completeness scores were gingival recession and dental implants, respectively.

Conclusions

The utilization of large language models has become increasingly prevalent, extending its applicability to patients within the healthcare domain. While ChatGPT may not offer absolute precision and comprehensive results without expert supervision, it is apparent that those within the field of periodontology can utilize it as an informational resource, albeit acknowledging the potential for inaccuracies.

## Introduction

Chat-Generative Pre-Trained Transformer (ChatGPT) is an artificial intelligence (AI) program that became available in November 2022 and creates text based on written prompts. This program has gained immense popularity for its web-based accessibility via OpenAI (OpenAI OpCo, LLC, San Francisco, CA). [1]. ChatGPT is a natural language processing model with 175 billion parameters that uses deep learning algorithms to generate human-like responses. As a versatile conversational agent, it can handle a variety of topics, making it useful for customer service, chatbots, and other applications [2]. ChatGPT offers next-generation models that can better combine clinical knowledge and interaction with dialogs. Its unique narrative interface enables innovative applications such as a patient simulation, a brainstorming tool, or a student companion [2]. Despite not being trained in any way, ChatGPT's performance that passes or is close to passing the United States Medical Licensing Examination (USMLE) shows its usability in the healthcare field. ChatGPT has a wide variety of uses in the field of dentistry. These include dentistry telemedicine services, supporting the clinical decisions made by the dentist, contributing to the education of dentistry students, and assisting in writing scientific articles, scientific evaluations, and patient information [3]. Large language model-based apps that have been tailored and refined have the potential to enhance dental telemedicine services [4]. In the near future, numerous language model programs are expected to possess the capability to efficiently acquire patient data, analyze symptoms, and generate preliminary diagnoses, which can subsequently be reviewed by a human dentist. Particularly in underprivileged areas with limited access to dental care, broad language models may prove more helpful in dental telemedicine services.

The advent of the internet and the subsequent development of search engines equipped with large language models have significantly influenced the accessibility of health-related patient information. By using ChatGPT, patients can find answers to their medical queries without having to go through numerous websites. In addition to having so many positive aspects, ChatGPT also has serious disadvantages. With the effect of ChatGPT's high self-confidence, it is quite difficult to confirm the accuracy and completeness of its information [3]. For users to trust this AI program, ChatGPT must perform similarly to humans in assessing medical knowledge and judgment [5]. ChatGPT is not suitable for use in support of clinical decisions due to the potential for misinformation or insufficient information and because it was not originally designed to provide medical guidance [6]. Therefore, the aim of this study is to evaluate the accuracy and completeness of the answers given by ChatGPT to the most frequently asked questions on different topics in the field of periodontology.

## Materials and methods

Study design and data source

The study group consisted of dental professionals specializing in periodontology in Turkey. Participation in the study was voluntary, and the consent of the individuals who agreed to participate in the study was obtained. In addition, the professional experience of all periodontology specialists who will make the evaluation was selected to be four to six years in the field of periodontology. In this way, the perspectives of the experts included in the study were standardized, as they had similar up-to-date knowledge in the field of periodontology. To ensure the calibration of the 20 selected periodontists for evaluating the responses, a systematic process is used. Prior to starting the evaluation, it was ensured that all 20 periodontists were familiar with the evaluation criteria and instructions. Training was given to those who were not familiar with it. A subject unrelated to the study topics, namely gingival enlargements, was selected for the purpose of conducting a training phase. During this phase, participants were instructed to assess and evaluate five different responses to questions pertaining to the chosen subject. The findings were subsequently deliberated together in order to detect any inconsistencies or disparities in their evaluations. Then, the evaluators were instructed to independently assess the responses to the questions by participating in many re-readings at their discretion over a period of one month.

The study was performed at Necmettin Erbakan University, Faculty of Dentistry, Konya, Turkey. The study was approved by the Necmettin Erbakan University, Faculty of Dentistry Scientific Research Ethics Committee (ref. 2023/294). The study was carried out in June 2023 using ChatGPT and was completed when the number of forms filled out by 20 periodontists was reached. No actual patient information was used in this study.

**Figure 1:**
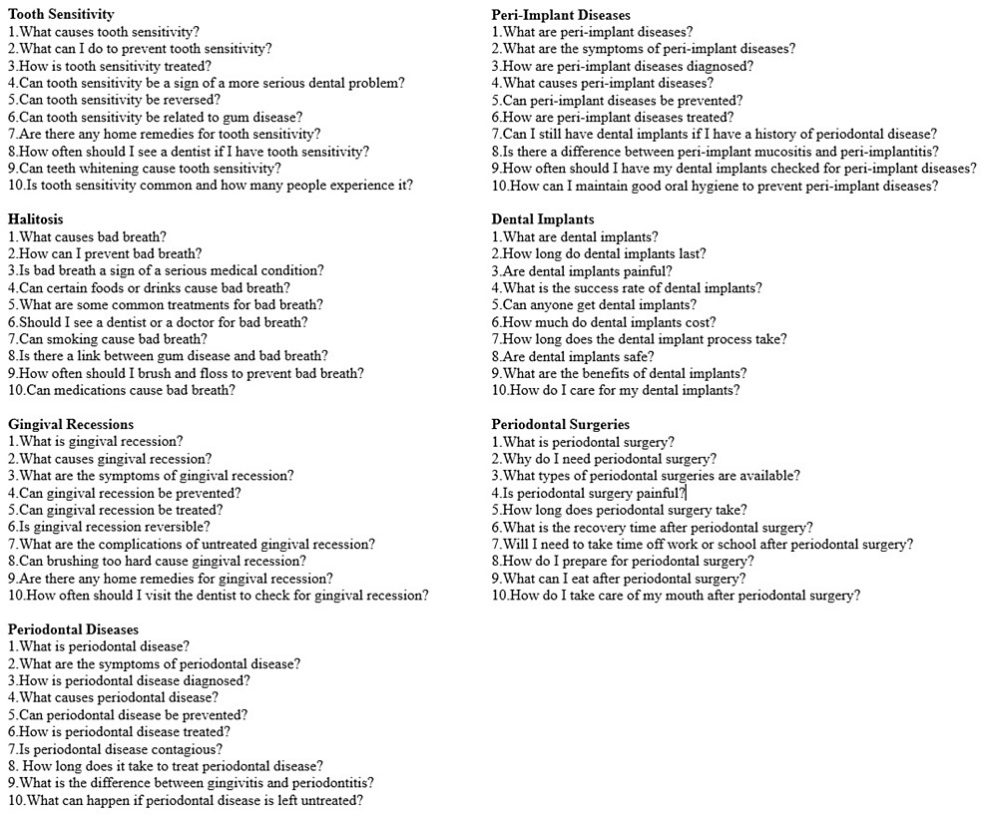
The questions asked on seven different topics within the field of periodontology.

Seven different main topics on which patients can ask basic questions about periodontology were determined by periodontists. The ChatGPT program was asked to create the 10 most frequently asked questions by patients about each of the seven main topics in the field of periodontology. These topics consist of periodontal diseases, peri-implant diseases, tooth sensitivity, gingival recessions, halitosis, dental implants, and periodontal surgery (Figure 1).

Each of the 70 questions in total created by ChatGPT was asked to be answered by ChatGPT by opening a new chat window and using the same current version of the ChatGPT program. All generated questions and their answers were recorded (Appendix A). Throughout the use of the ChatGPT program, only the English language was used. Experienced periodontists were contacted via email to evaluate the answers given by ChatGPT. Covariates of the periodontologists were recorded, including their gender, age, professional experience, and previous experience with artificial intelligence.

Evaluation of the responses to each question

Accuracy and completeness were assessed with separate Likert scales for each question [7]. Before the examination, the experts were instructed to assess the completeness of the response before assessing its accuracy.

The Accuracy Likert Scale

1: completely incorrect; 2: more incorrect than correct; 3: approximately equal correct and incorrect; 4: more correct than incorrect; 5: nearly all correct; 6: correct.

The Completeness Likert Scale

1: incomplete; addresses some aspects of the question, but significant parts are missing or incomplete; 2: adequate, addresses all aspects of the question, and provides the minimum amount of information required to be considered complete; 3: comprehensive, addresses all aspects of the question, and provides additional information or context beyond what was expected.

Statistical analysis

The data were evaluated using the IBM SPSS software version 23.0 (IBM Corp., Armonk, NY). The sample size of the study was within the 90% confidence interval, the marginal error was calculated as 0.05, and a total of 20 people were included in the study. Whether the data were normally distributed or not was examined with the Kolmogorov-Smirnov test. The Mann-Whitney U test and Kruskal-Wallis H test were used in the analysis of quantitative variables, and the chi-square test was used in the analysis of qualitative variables. The relationship between quantitative variables was evaluated by logistic regression analysis. Spearman's rho correlation coefficients were used to examine relationships between accuracy scores and completeness scores. A P-value <.05 was accepted as statistically significant.

## Results

A total of 70 questions were created by ChatGPT, 10 questions from each, related to seven main periodontology subjects, and the answers to each of them are presented in Appendix A. The gender, age, and AI usage experiences of 20 periodontology specialists participating in the study are given in Table 1.

**Table 1:** Demographic data of experts in the field of periodontology evaluating the answers given by ChatGPT SD: standard deviation

	Age (years)			Artificial intelligence experience		
Gender	Mean	SD	P-value	Yes	No	P-value
Female (N=12)	31.83	3.88	0.564	1	11	0.255
Male (N=8)	32.75	3.05		3	5	

There was no statistically significant difference between the use of artificial intelligence by gender and age.

The distribution of accuracy and completeness scores on the Likert scale of the answers given to the questions according to topic headings is presented in Table 2.

**Table 2:** Distribution of accuracy and completeness scores for subjects Data are represented as n (%).

	Accuracy scores							Completeness scores		
	Completely incorrect (1)	More incorrect than correct (2)	Approximately equal correct and incorrect (3)	More correct than incorrect (4)	Nearly all correct (5)	Correct (6)		Incomplete (1)	Adequate (2)	Comprehensive (3)
Periodontal disease	0 (0)	0 (0)	0 (0)	5 (2.5)	74 (37)	121 (60.5)		13 (6.5)	114 (57)	73 (36.5)
Peri-implant disease	0 (0)	0 (0)	0 (0)	10 (5)	48 (24)	142 (71)		23 (11.5)	94 (47)	83 (41.5)
Tooth sensitivity	0 (0)	8 (4)	0 (0)	12 (6)	61 (30.5)	119 (59.5)		24 (12)	80 (40)	96 (48)
Gingival recession	0 (0)	7 (3.5)	5 (2.5)	18 (9)	86 (43)	84 (42)		30 (15)	96 (48)	74 (37)
Halitosis	0 (0)	0 (0)	4 (2)	4 (2)	47 (23.5)	145 (72.5)		16 (8)	95 (47.5)	89 (44.5)
Dental implants	0 (0)	4 (2)	0 (0)	14 (7)	72 (36)	110 (55)		22 (11)	114 (57)	64 (32)
Periodontal surgery	0 (0)	0 (0)	0 (0)	8 (4)	66 (33)	126 (63)		14 (7)	114 (57)	72 (36)

Each of the answers to the periodontology questions that could be asked by the patients was evaluated for accuracy and completeness. Table 3 presents the statistical measures of the scores obtained in the seven subjects, including the median, mode, and mean values.

**Table 3:** Accuracy and completeness scores for AI-generated answers to questions on key topics in periodontology SD: standard deviation

		Accuracy	Completeness	P-value
Periodontal disease	Median (min.-max.)	6 (4-6)	2 (1-3)	<0.001
	Mode	6	2	
	Mean±SD	5.59 ± .19	2.31 ± .2	
Peri-implant disease	Median (min.-max.)	6 (4-6)	2 (1-3)	<0.001
	Mode	6	2	
	Mean±SD	5.66 ± .23	2.29 ± .36	
Tooth sensitivity	Median (min.-max.)	6 (2-6)	2 (1-3)	<0.001
	Mode	6	3	
	Mean±SD	5.41 ± .31	2.36 ± .23	
Gingival recession	Median (min.-max.)	5 (2-6)	2 (1-3)	<0.001
	Mode	5	2	
	Mean±SD	5.21 ± .43	2.61 ± .25	
Halitosis	Median (min.-max.)	6 (3-6)	2 (1-3)	<0.001
	Mode	6	2	
	Mean±SD	5.67 ± .31	2.37 ± .34	
Dental implants	Median (min.-max.)	6 (2-6)	2 (1-3)	<0.001
	Mode	6	2	
	Mean±SD	5.42 ± .07	2.21 ± .23	
Periodontal surgery	Median (min.-max.)	6 (4-6)	2 (1-3)	<0.001
	Mode	6	2	
	Mean±SD	5.59 ± .31	2.29 ± .24	

Among the seven main topics we chose in periodontology, halitosis had the highest accuracy score (5.67±0.31), while the lowest accuracy was found for gingival recessions (5.21±0.43). When the completeness scores were evaluated among the subjects, the highest score was in gingival recession (2.61±0.25), while the lowest score was in dental implants (2.21±0.23).

The differences in accuracy and completeness between subjects were evaluated with the Kruskal-Wallis test. Accuracy and completeness among all subjects were evaluated separately with the Mann-Whitney U test for statistical differences.

A statistically significant difference was observed in both accuracy and completeness scores during the inter-subject examination, with a P-value of less than 0.05. The accuracy of the answers given to the topic of gingival recession was statistically lower than all other topics, except for "tooth sensitivity" and "dental implants" (P<0.05). There was no statistically significant difference in accuracy when comparing the subjects of "gingival recession," "tooth sensitivity," and "dental implants" (P>0.05). The statistical analysis revealed that the score of completeness in the responses provided for the subject of gingival recession was significantly higher compared to all other subjects, with the exceptions of "tooth sensitivity" and "halitosis" (P<0.05). There was no statistically significant difference in terms of completeness between the subjects "gingival recession", "tooth sensitivity", and "halitosis" (P>0.05).

There was a moderate correlation in accuracy and completeness scores among all subjects (Spearman’s r=0.46, 95% CI 0.3 to 0.5, P<0.01).

## Discussion

Given the public accessibility of ChatGPT, it is clear that patients can often ask questions to large language models about their medical condition in the first stage. Our study focused on the most frequently asked questions by patients and the quality of the answers given by artificial intelligence. To the best of our knowledge, this study is the first in the field of periodontology to evaluate the information provided by artificial intelligence chatbots. For this reason, there is no study to compare the numerical data in the results.

With the development of technology, the first place that patients ask about a subject they are curious about is the internet. Various studies have been conducted on the information accuracy and quality of search engines on the Internet and videos on YouTube [8]. A recent study by Beaumont et al. investigated framing in online patient information for people newly diagnosed with periodontitis [9]. Their work was a cross-sectional analysis of websites that came up with a Google search for the term 'gum disease' using linguistic techniques. The results showed that the fear of patients due to negative framing can increase the desire to be treated and self-care. However, the risks of patient information systems were emphasized by showing that negative situations could frighten and demotivate the patient even more. Bizzi et al. evaluated the quality of information available on the Web about gum disease according to the Journal of the American Medical Association criteria [10]. As a result, they showed that Google can use sites with high-quality information for patient information purposes [10].

Numerous studies have been conducted in the field of health, especially in medical education, in which the performance of ChatGPT was evaluated [11, 12]. Measuring AI medical knowledge compared to specialist clinicians is a critical step in assessing these qualifications. In a study by Kung et al., the performance of ChatGPT, a language-based AI, in the United States Medical Licensing Examination (USMLE) was evaluated [13]. ChatGPT was found to perform at or near the 60% accuracy threshold on this exam, which is required for obtaining a medical license in the United States [13, 14]. The fact that an AI program reaches this criterion without any special training shows that AI developments have come to an extremely advanced level. Huh's study aimed to compare the interpretation and knowledge of ChatGPT compared to medical students in Korea [5]. Their study explained that ChatGPT's ability to understand and interpret parasitology exam questions is still not at the same level as that of Korean medical students. On the other hand, Gilson et al. aimed to investigate and observe ChatGPT's performance in the USMLE [15]. The authors reported that the model performed over 60% on the National Board of Medical Examiners (NBME)-Free-Step-1 dataset, achieving a passing score for a medical student in the third year [15]. Sabry et al. analyzed ChatGPT using a clinical toxicology case of acute organophosphate poisoning. As reported by the authors, ChatGPT was able to answer all questions regarding the case introduced [16]. Yeo et al. examined the accuracy and reproducibility of ChatGPT when answering questions about information, disease management, and emotional support for cirrhosis and hepatocellular carcinoma [17]. ChatGPT's answers to 164 questions were evaluated by two transplant hepatologists and one reviewer. As a result, it has been shown that ChatGPT may have a role as an additional information tool for patients and physicians to improve outcomes [17].

Although it has been demonstrated in numerous studies that ChatGPT is unsuccessful in providing technical responses, it is effective in providing replies to inquiries intended to provide information to patients [7]. There are very few studies that have been published in the literature that corroborate the findings of our research [18].

Johnson et al. In a study they conducted, ChatGPT's answers to 284 medical questions in 17 different specialties were evaluated by experts [7]. They evaluated with two different Likert scales on accuracy and completeness. The mean accuracy score for all questions in the study (n=284) was 5.5, which was between almost completely and completely correct, and the median completeness score was three, which was complete and comprehensive [7].

In a study conducted by Balel, questions about oral and maxillofacial surgery, which were created by the author himself, were asked to ChatGPT, and the answers given by ChatGPT were evaluated by oral, dental, and maxillofacial surgery specialists [18]. As a result, ChatGPT's answers to patient-focused questions were found to be accurate and helpful by experts [18]. In addition, they did not find a statistical difference between the topics in oral and maxillofacial surgery. Similarly, in our study, although the answers given by ChatGPT were "nearly all correct" in terms of accuracy, statistical differences were found between the subjects in terms of accuracy. The topic of gingival recessions had the highest completeness score in terms of information, although it had the lowest accuracy score. This indicates that the greater the amount of information ChatGPT gives, the greater the risk of giving false information.

It is also useful to draw attention to some points regarding the questions in our study. The answers to some of the questions asked on some specific topics, such as "dental implants and peri-implant diseases," are still controversial or unknown in the literature. The following five examples of these questions can be given: 1. How long do dental implants last? 2. What is the success rate of dental implants? 3. What causes peri-implant diseases? 4. Can peri-implant diseases be prevented? 5. How are peri-implant diseases treated?

The answers to these questions are still being investigated by various studies, and the answers are not clear. However, it was included in our study because it was thought that patients could also ask these questions. When ChatGPT's answers to these questions were examined, it was observed that ChatGPT also said that there were no definitive answers to the questions. ChatGPT stated that how long dental implants can remain in the mouth or their success rate may depend on many factors and did not provide clear information. Although there are debates in the literature, more definitive answers have been given to questions about the causes of peri-implant diseases, how they can be prevented, and how they are treated. Therefore, it is essential to conduct further research on large language models such as ChatGPT to evaluate the reliability of information in the field of healthcare.

The present study is subject to multiple limitations. One of the limitations of our study is the small number of periodontology professionals who assessed the responses. Furthermore, the scope of our study was limited to the examination of queries related exclusively to the field of periodontology in dentistry, which restricts the overall applicability of our evaluation to ChatGPT.

## Conclusions

Artificial intelligence technology is developing very rapidly. Even though ChatGPT cannot provide 100% accurate and comprehensive findings without expert oversight, it is evident that patients in the field of periodontology can still use it for informational purposes by accepting some error risks. There is a need for more research on the application of AI chatbots in the field of periodontology. This involves the inclusion of additional assessors and an examination of different question forms.

## Appendices

Appendix A

A.  *Tooth Sensitivity*

1.     What causes tooth sensitivity?

Tooth sensitivity can be caused by a variety of factors, including:

Enamel erosion: The outer layer of your teeth, called enamel, can wear down over time due to factors such as brushing too hard or consuming acidic foods and drinks.

Gum recession: When your gum tissue starts to pull back from your teeth, it exposes the sensitive root surface of your teeth.

Tooth decay: Cavities can develop in your teeth and cause sensitivity when they reach the nerve inside.

Cracked or chipped teeth: When the protective outer layer of your teeth is damaged, it can expose the inner layers to stimuli that cause sensitivity.

Teeth grinding: The repeated grinding or clenching of teeth can wear away the enamel and cause sensitivity.

Whitening treatments: Some tooth whitening treatments can cause sensitivity in some individuals.

Dental procedures: Certain dental procedures, such as fillings or root canals, can cause temporary sensitivity.

2. What can I do to prevent tooth sensitivity?

Tooth sensitivity can be prevented or minimized by following these tips:

Brush and floss regularly: Regularly brushing and flossing your teeth helps prevent the buildup of plaque and tartar that can cause tooth sensitivity.

Use a soft-bristled toothbrush: A hard-bristled toothbrush can cause enamel erosion and gum recession, leading to tooth sensitivity.

Use toothpaste for sensitive teeth: Toothpaste designed for sensitive teeth contains ingredients that help block the nerve endings in the teeth, reducing sensitivity.

Avoid acidic foods and drinks: Acidic foods and drinks can erode tooth enamel, leading to sensitivity. Limit your intake of citrus fruits, sodas, and other acidic foods and drinks.

Use fluoride mouthwash: Fluoride strengthens tooth enamel, making it more resistant to acid and plaque.

Wear a mouthguard: If you grind your teeth at night, wearing a mouthguard can help prevent enamel erosion and reduce tooth sensitivity.

Visit your dentist regularly: Regular dental checkups can help identify and treat any dental problems before they lead to sensitivity.

3. How is tooth sensitivity treated?

Tooth sensitivity can be treated in several ways, depending on the cause and severity of the sensitivity. Some possible treatments include:

Desensitizing toothpaste: Special toothpaste containing compounds such as potassium nitrate, strontium chloride or fluoride, can help to reduce sensitivity over time.

Fluoride treatments: A dentist can apply fluoride gel or varnish to the sensitive areas of the teeth to strengthen tooth enamel and reduce sensitivity.

Dental bonding: If the sensitivity is caused by exposed tooth roots or small cracks or chips, dental bonding can be used to cover and protect the affected area.

Dental sealants: If the sensitivity is caused by receding gums, dental sealants can be applied to the exposed tooth root to protect it from further damage.

Root canal therapy: In cases where the sensitivity is caused by deep decay or infection, a root canal procedure may be necessary to remove the infected tissue and alleviate the sensitivity.

It's important to see a dentist to determine the cause of tooth sensitivity and receive appropriate treatment. Additionally, practicing good oral hygiene habits such as brushing twice a day and flossing daily can help prevent tooth sensitivity from developing or worsening.

4. Can tooth sensitivity be a sign of a more serious dental problem?

Tooth sensitivity can be a symptom of a more serious dental problem in some cases. Tooth sensitivity occurs when the enamel on the teeth wears down, exposing the underlying dentin and nerves, which can cause pain or discomfort when the teeth come into contact with hot, cold, sweet, or acidic substances.

Some potential causes of tooth sensitivity that may indicate a more serious dental problem include:

Tooth decay: If tooth sensitivity is accompanied by a persistent toothache or visible pits or holes in the teeth, it could be a sign of tooth decay, which can lead to more serious dental problems if left untreated.

Gum disease: Receding gums can expose the roots of the teeth, which can lead to tooth sensitivity. If left untreated, gum disease can progress and lead to tooth loss.

Cracked or broken teeth: Teeth that are cracked or broken can also lead to tooth sensitivity. If left untreated, a cracked or broken tooth can become infected or lead to more serious dental problems.

Bruxism: Grinding or clenching the teeth can wear down the enamel and cause tooth sensitivity. Bruxism can also lead to other dental problems, such as jaw pain and headaches.

If you are experiencing tooth sensitivity, it's important to see a dentist to determine the underlying cause and develop a treatment plan to address the issue.

5. Can tooth sensitivity be reversed?

Tooth sensitivity can often be managed and reduced, but it may not always be possible to completely reverse it, especially in cases of advanced tooth decay or gum disease. However, there are several things you can do to reduce sensitivity and prevent further damage to your teeth:

Use a toothpaste specifically designed for sensitive teeth: These toothpastes contain ingredients such as potassium nitrate or strontium chloride that can help block the tiny tubes in the dentin that allow sensitivity.

Avoid acidic foods and drinks: Acidic foods and drinks can erode tooth enamel, making teeth more sensitive. Limit your consumption of acidic foods and drinks such as citrus fruits, soda, and sports drinks.

Practice good oral hygiene: Brush your teeth twice a day with a soft-bristled toothbrush and fluoride toothpaste. Floss daily to remove plaque and food particles from between your teeth.

Use a mouthguard: If you grind your teeth at night, wearing a mouthguard can help reduce sensitivity and protect your teeth from further damage.

See your dentist regularly: Regular dental check-ups can help identify and treat any dental problems that may be causing sensitivity, such as tooth decay or gum disease.

If your tooth sensitivity persists despite these measures, it's important to see your dentist to determine the underlying cause and develop a treatment plan. In some cases, procedures such as fillings, crowns, or root canals may be necessary to address the underlying problem and reduce sensitivity.

6. Can tooth sensitivity be related to gum disease?

Yes, tooth sensitivity can be related to gum disease. Gum disease, also known as periodontal disease, is a common condition that affects the gums and bones surrounding the teeth. It can cause inflammation and damage to the gum tissue, which can lead to gum recession and exposure of the sensitive root surfaces of the teeth.

When the roots of the teeth become exposed, they can be more sensitive to hot, cold, sweet, and acidic foods and drinks. This can cause discomfort or pain when eating or drinking certain things. In addition, gum disease can cause the formation of pockets between the teeth and gums, which can trap food particles and bacteria, leading to further inflammation and sensitivity.

If you are experiencing tooth sensitivity, it is important to see a dentist to determine the underlying cause and receive appropriate treatment. Depending on the severity of your gum disease, your dentist may recommend a deep cleaning procedure called scaling and root planing, antibiotics, or even surgery in some cases. Proper oral hygiene, including brushing twice a day and flossing daily, can also help prevent gum disease and reduce tooth sensitivity.

7. Are there any home remedies for tooth sensitivity?

Yes, there are several home remedies that can help with tooth sensitivity. Here are a few:

Saltwater rinse: Mix a teaspoon of salt with a glass of warm water and swish it around your mouth for 30 seconds before spitting it out. This can help reduce inflammation and sensitivity.

Clove oil: Apply a small amount of clove oil directly to the affected tooth or gum area with a cotton swab. Clove oil has natural numbing properties that can help relieve pain.

Green tea: Brew a cup of green tea and swish it around in your mouth for a few minutes before spitting it out. Green tea contains compounds that can help reduce inflammation and bacteria in the mouth.

Honey and warm water: Mix a teaspoon of honey with a glass of warm water and drink it slowly. Honey has natural antibacterial properties that can help fight off harmful bacteria in the mouth.

Coconut oil pulling: Swish a tablespoon of coconut oil in your mouth for 15-20 minutes before spitting it out. This can help reduce inflammation and kill harmful bacteria in the mouth.

However, it's important to note that these home remedies may only provide temporary relief and may not treat the underlying cause of your tooth sensitivity. If your tooth sensitivity persists or worsens, it's best to consult a dentist for a proper diagnosis and treatment plan.

8. How often should I see a dentist if I have tooth sensitivity?

If you are experiencing tooth sensitivity, it is recommended that you visit a dentist as soon as possible to determine the underlying cause and receive appropriate treatment. Depending on the severity of your sensitivity and the cause, your dentist may recommend more frequent dental check-ups to monitor the condition and ensure that it is properly managed.

In general, it is recommended that individuals visit the dentist at least twice a year for routine dental check-ups and cleanings, regardless of whether they have tooth sensitivity or not. However, if you have a history of dental problems or conditions that increase your risk of tooth sensitivity, your dentist may recommend more frequent visits.

It's important to remember that tooth sensitivity can be a symptom of a more serious underlying dental problem, such as tooth decay, gum disease, or a cracked tooth. Ignoring the symptoms of tooth sensitivity can lead to more serious dental problems and potentially require more extensive treatment. Therefore, it's essential to seek the advice of a dental professional if you are experiencing any tooth sensitivity.

9. Can teeth whitening cause tooth sensitivity?

Yes, teeth whitening can cause tooth sensitivity in some people. The most common type of tooth sensitivity associated with teeth whitening is called "bleaching sensitivity" and is usually temporary.

The active ingredient in most teeth whitening products is hydrogen peroxide or carbamide peroxide, which can penetrate the enamel and reach the dentin layer of the tooth. This can cause temporary irritation and sensitivity, particularly if the whitening treatment is overused or if the concentration of the whitening agent is too high.

To minimize the risk of tooth sensitivity, it's important to follow the instructions provided with the teeth whitening product and to avoid overusing the product. It's also a good idea to use toothpaste designed for sensitive teeth, which can help to desensitize the teeth and reduce discomfort.

If you experience tooth sensitivity after teeth whitening, it's usually temporary and will go away on its own within a few days or weeks. However, if the sensitivity persists or is particularly severe, it's important to consult your dentist, as there may be an underlying issue that needs to be addressed.

10. Is tooth sensitivity common and how many people experience it?

Tooth sensitivity is a relatively common dental issue, and many people experience it at some point in their lives. According to a study published in the Journal of the American Dental Association, about 1 in 8 adults in the United States have sensitive teeth, which amounts to around 12% of the population.

Tooth sensitivity can be caused by a variety of factors, such as tooth decay, gum recession, enamel erosion, tooth grinding, and more. It is characterized by a sharp, shooting pain or discomfort in the teeth when they are exposed to hot, cold, sweet, or acidic foods and drinks, or when pressure is applied to them.

Fortunately, tooth sensitivity can often be treated or managed by practicing good oral hygiene habits, using desensitizing toothpaste or mouthwash, avoiding certain triggers, and seeing a dentist for professional treatment if necessary.

B. *Halitosis*

1. What causes bad breath?

Bad breath, also known as halitosis, can be caused by various factors. Some of the most common causes of bad breath include:

Poor oral hygiene: Failing to brush and floss regularly can lead to the buildup of food particles and bacteria in the mouth, which can cause bad breath.

Food and drink: Certain foods and drinks, such as garlic, onions, coffee, and alcohol, can cause bad breath.

Dry mouth: Saliva helps to wash away food particles and bacteria in the mouth. When there is not enough saliva, such as in the case of dry mouth, bad breath can occur.

Smoking and tobacco use: Tobacco use can cause a distinct and unpleasant odor in the mouth, as well as other health problems.

Medical conditions: Certain medical conditions, such as sinus infections, respiratory tract infections, and acid reflux can cause bad breath.

Medications: Some medications can cause dry mouth or have a chemical odor that can cause bad breath.

Poor diet: Eating a diet high in sugary and processed foods can lead to bad breath as bacteria feed on the sugars and produce unpleasant odors.

If you are concerned about persistent bad breath, it is important to speak to a healthcare provider, who can help identify the underlying cause and recommend appropriate treatment.

2. How can I prevent bad breath?

There are several things you can do to prevent bad breath:

Practice good oral hygiene: Brush your teeth at least twice a day and floss once a day to remove food particles and plaque that can cause bad breath.

Scrape your tongue: Use a tongue scraper to remove bacteria and dead cells from your tongue. This can significantly reduce bad breath.

Stay hydrated: Drink plenty of water throughout the day to keep your mouth moist and prevent dry mouth, which can cause bad breath.

Chew sugarless gum or suck on sugarless candy: Chewing gum or sucking on candy stimulates the production of saliva, which can help wash away bacteria and food particles that cause bad breath.

Avoid foods that can cause bad breath: Foods such as garlic, onions, and spicy foods can cause bad breath. Try to avoid them or brush your teeth and tongue after consuming them.

Quit smoking: Smoking can cause bad breath, as well as other oral health problems. Quitting smoking can improve your overall health and prevent bad breath.

If your bad breath persists despite taking these measures, it may be a sign of an underlying health problem, and you should consult a dentist or a doctor.

3. Is bad breath a sign of a serious medical condition?

Bad breath, also known as halitosis, can be caused by a number of factors, including poor dental hygiene, certain foods and drinks, smoking, and dry mouth. In most cases, bad breath is not a sign of a serious medical condition.

However, there are some medical conditions that can cause bad breath. For example, gum disease, cavities, and oral infections can all lead to bad breath. Additionally, some medical conditions that affect the respiratory system, such as sinus infections, bronchitis, and pneumonia, can cause bad breath.

In rare cases, bad breath can be a sign of a more serious medical condition, such as liver or kidney disease, diabetes, or certain cancers. However, these conditions usually have other symptoms in addition to bad breath.

If you are concerned about your bad breath or if it is accompanied by other symptoms, it is a good idea to speak with your healthcare provider. They can help determine the cause of your bad breath and recommend appropriate treatment.

4. Can certain foods or drinks cause bad breath?

Yes, certain foods and drinks can cause bad breath. This is because some foods and drinks contain volatile compounds that can be released into the breath as they are digested. These compounds can be strong-smelling and can linger in the mouth and on the tongue, causing bad breath.

Some common culprits include garlic, onions, spicy foods, alcohol, coffee, and acidic foods and drinks like citrus fruits and vinegar. Dairy products can also cause bad breath if they are not properly digested, as this can lead to the buildup of bacteria in the mouth.

In addition to these foods and drinks, poor dental hygiene and certain medical conditions can also cause bad breath. If you are concerned about your breath, it is a good idea to see a dentist or doctor to rule out any underlying health issues.

5. What are some common treatments for bad breath?

There are several common treatments for bad breath, including:

Practice good oral hygiene: Brushing and flossing your teeth at least twice a day can help remove food particles and plaque that can cause bad breath.

Use mouthwash: Using a mouthwash or rinse can help kill bacteria in your mouth and freshen your breath.

Stay hydrated: Drinking plenty of water can help keep your mouth moist and prevent dry mouth, which can contribute to bad breath.

Avoid tobacco and alcohol: Tobacco and alcohol can contribute to bad breath, so it's best to avoid them altogether.

Chew gum or mints: Chewing gum or mints can help stimulate saliva production and freshen your breath.

Visit your dentist: Regular dental check-ups can help identify and treat any underlying dental issues that may be contributing to your bad breath.

Consider changing your diet: Certain foods, such as onions and garlic, can contribute to bad breath, so reducing your intake of these foods may help.

It's important to note that if your bad breath persists despite these treatments, it's best to consult a healthcare professional to rule out any underlying medical conditions.

6. Should I see a dentist or a doctor for bad breath?

If you are experiencing bad breath, it is recommended that you first see a dentist. Dental problems, such as gum disease or tooth decay, are a common cause of bad breath. A dentist can examine your mouth, teeth, and gums to determine if there are any issues that need to be addressed. Additionally, they can provide you with guidance on proper oral hygiene practices and recommend products to help combat bad breath.

However, if the bad breath is persistent and not related to any dental issues, it may be necessary to see a doctor to rule out any underlying medical conditions, such as sinus infections, diabetes, or gastrointestinal problems. In this case, your doctor can perform a thorough evaluation and recommend appropriate treatment.

7. Can smoking cause bad breath?

Yes, smoking can cause bad breath. When a person smokes, the chemicals in the tobacco can stick to the mouth, throat, and lungs. This can cause a persistent bad smell, commonly known as smoker's breath. In addition, smoking can dry out the mouth, which reduces the amount of saliva produced. Saliva helps to wash away bacteria and food particles in the mouth, so when there is less saliva, it can lead to an increase in bacteria growth, which can also cause bad breath. Quitting smoking can help improve oral health and reduce the risk of bad breath.

8. Is there a link between gum disease and bad breath?

Yes, there is a link between gum disease and bad breath. Gum disease, also known as periodontal disease, is caused by bacteria that accumulate in the mouth and form plaque on the teeth and gums. This bacteria can produce foul-smelling gases that can cause bad breath or halitosis.

In addition, gum disease can cause the gums to become inflamed and bleed, which can also contribute to bad breath. The inflammation and bleeding can create pockets between the teeth and gums, allowing more bacteria to grow and cause further bad breath.

If you have persistent bad breath, it's a good idea to see a dentist or periodontist to rule out gum disease as a possible cause. Proper oral hygiene, including brushing twice a day, flossing daily, and regular dental checkups, can help prevent gum disease and keep your breath fresh.

9. How often should I brush and floss to prevent bad breath?

To prevent bad breath, it's recommended that you brush your teeth at least twice a day, preferably after meals. You should also floss at least once a day, preferably before going to bed. Brushing and flossing help to remove food particles and bacteria from your mouth, which can cause bad breath.

In addition to regular brushing and flossing, it's also important to clean your tongue regularly. The tongue can harbor bacteria and food particles, which can contribute to bad breath. You can clean your tongue using a tongue scraper or by gently brushing your tongue with your toothbrush.

If you find that you still have bad breath despite following a regular oral hygiene routine, you may want to consult with your dentist or healthcare provider. They can help you identify any underlying conditions that may be contributing to your bad breath, such as gum disease or digestive issues, and recommend appropriate treatment.

10. Can medications cause bad breath?

Yes, some medications can cause bad breath as a side effect. This is because certain medications can affect the natural balance of bacteria in the mouth or produce chemicals that have an odor. Some examples of medications that can cause bad breath include:

Antidepressants: Some antidepressants can cause dry mouth, which can lead to bad breath.

Antihistamines: Antihistamines can also cause dry mouth and reduce saliva production, leading to bad breath.

Blood pressure medications: Some blood pressure medications, such as nifedipine and diltiazem, can cause bad breath as a side effect.

Diabetes medications: Some diabetes medications can produce chemicals called ketones, which can cause a fruity or acetone-like odor in the breath.

Pain medications: Opioid pain medications can cause dry mouth and reduce saliva production, leading to bad breath.

If you are experiencing bad breath as a side effect of your medication, it's important to speak with your doctor. They may be able to adjust your medication or suggest ways to manage the side effects, such as drinking more water or using sugar-free gum or lozenges to stimulate saliva production.

C. *Gingival Recession*

1. What is gingival recession?

Gingival recession, also known as receding gums, is a condition where the gum tissue that surrounds and protects the teeth begins to pull away or wear down, exposing more of the tooth or even the tooth's root. This can lead to sensitivity, discomfort, and even tooth loss if left untreated. Gingival recession is often caused by factors such as gum disease, aggressive brushing or flossing, poor oral hygiene, genetics, or even hormonal changes. Treatment options may include scaling and root planing, gum grafting, or other procedures to restore gum tissue and protect the tooth.

2. What causes gingival recession?

Gingival recession, also known as receding gums, is typically caused by a combination of several factors, including:

Periodontal diseases: Gum disease, which is caused by the buildup of plaque and tartar on teeth, can damage the gums and lead to gingival recession.

Aggressive brushing: Brushing too hard or using a toothbrush with hard bristles can wear away the gum tissue and cause it to recede.

Genetics: Some people are more predisposed to developing gingival recession due to their genetics.

Poor dental hygiene: Neglecting oral hygiene can lead to the buildup of plaque and tartar, which can contribute to gingival recession.

Hormonal changes: Changes in hormone levels during puberty, pregnancy, and menopause can make gums more sensitive and prone to recession.

Tobacco use: Smoking or using other tobacco products can lead to gum disease and gum recession.

Misaligned teeth: Teeth that are not properly aligned can put undue pressure on certain parts of the gums, leading to recession.

Trauma or injury: Injury to the mouth or gums, such as from sports or accidents, can cause gum recession.

If you are experiencing gingival recession, it is important to consult with a dentist or periodontist to determine the underlying cause and receive appropriate treatment.

3. What are the symptoms of gingival recession?

Gingival recession refers to the gradual loss of gum tissue, which can expose the roots of the teeth and lead to sensitivity, discomfort, and even tooth decay. Some common symptoms of gingival recession include:

Tooth sensitivity: Exposed tooth roots can cause increased sensitivity to hot, cold, sweet, or sour foods and drinks.

Long or uneven teeth: When gums recede, teeth may appear longer or uneven.

Visible tooth roots: As gums recede, the tooth roots become visible and may have a yellowish or darker color than the rest of the tooth.

Tooth mobility: As gum tissue recedes, the teeth may become loose or shift positions.

Gum inflammation and bleeding: Receding gums can leave the tooth root exposed and more prone to inflammation and bleeding.

If you notice any of these symptoms, it is important to consult with a dentist or periodontist for an evaluation and appropriate treatment.

4. Can gingival recession be prevented?

Gingival recession, which is the gradual loss of gum tissue around teeth, can sometimes be prevented with good oral hygiene practices and regular dental checkups. Here are some ways to help prevent gingival recession:

Brush and floss regularly: Brush your teeth twice a day, and floss at least once a day. This can help remove plaque and bacteria that can irritate the gums and cause them to recede.

Use a soft-bristled toothbrush: Hard-bristled toothbrushes can be too abrasive and cause damage to the gum tissue. A soft-bristled toothbrush is gentler and less likely to cause gum recession.

Use proper brushing technique: Use gentle circular motions when brushing, and avoid scrubbing back and forth or side to side, which can cause gum irritation.

Avoid tobacco products: Smoking and chewing tobacco can increase the risk of gum disease, which is a leading cause of gum recession.

Eat a healthy diet: A diet that is high in sugary or acidic foods can contribute to gum disease and other oral health problems. Eating a diet that is rich in fruits, vegetables, and whole grains can help maintain good oral health.

Visit your dentist regularly: Regular dental checkups can help identify and treat gum disease and other oral health problems before they become more serious.

Address teeth grinding: If you grind your teeth, talk to your dentist about getting a mouthguard to protect your teeth and gums from damage.

While these practices can help reduce the risk of gingival recession, it's important to remember that some factors, such as genetics and age, can also contribute to gum recession. If you notice any signs of gum recession, such as tooth sensitivity or longer-looking teeth, talk to your dentist about treatment options.

5. Can gingival recession be treated?

Yes, gingival recession can be treated. The treatment for gingival recession depends on the severity of the recession, the underlying cause, and the patient's oral health.

If the recession is mild, it may be treated with a thorough cleaning of the teeth and gums, followed by regular dental visits and good oral hygiene practices. This can help to prevent further recession and promote the growth of healthy gum tissue.

In more severe cases, treatment may involve surgical procedures such as gum grafting or guided tissue regeneration. These procedures involve the transplantation of gum tissue or the regeneration of bone and tissue around the tooth to promote the growth of healthy gum tissue.

It's important to note that the success of treatment for gingival recession depends on the underlying cause of the recession, the severity of the condition, and the patient's commitment to maintaining good oral hygiene practices. Therefore, it's recommended to consult with a dental professional to determine the most appropriate treatment plan for your individual case.

6. Is gingival recession reversible?

Gingival recession, also known as receding gums, occurs when the gum tissue that surrounds the teeth wears away, exposing the tooth root. While there are treatments available to address the issue, gingival recession is generally considered irreversible.

There are various factors that can contribute to gingival recession, including gum disease, aggressive tooth brushing, genetics, hormonal changes, and trauma to the gums. The appropriate treatment for gingival recession depends on the underlying cause and severity of the condition.

Some treatments for gingival recession include scaling and root planing (deep cleaning), gum grafting (where tissue is taken from another part of the mouth and used to cover the exposed root), and orthodontic treatment to reposition the teeth.

While these treatments can help to prevent further recession and improve the appearance of the gums, they generally cannot reverse the damage that has already been done. It is important to practice good oral hygiene habits and visit a dentist regularly to prevent further progression of gingival recession.

7. What are the complications of untreated gingival recession?

Untreated gingival recession can lead to several complications, including:

Tooth sensitivity: When the gum tissue recedes, the root of the tooth becomes exposed, which can lead to increased sensitivity to hot, cold, or sweet foods and beverages.

Tooth decay: The root of the tooth is more vulnerable to decay than the enamel, which can increase the risk of cavities.

Gum disease: Receding gums can make it easier for bacteria to enter the tooth and cause gum disease, which can result in further gum recession and tooth loss.

Tooth loss: As the gum tissue recedes, it can expose more of the tooth, which can weaken the tooth's structure and lead to tooth loss.

Aesthetic concerns: Receding gums can make the teeth look longer than usual, which can cause aesthetic concerns and make a person feel self-conscious about their smile.

It is important to seek dental treatment if you are experiencing gum recession to prevent these complications from occurring.

8. Can brushing too hard cause gingival recession?

Yes, brushing too hard or using a toothbrush with hard bristles can cause gingival recession. Gingival recession is the process in which the gum tissue pulls back or wears away from the tooth, exposing the tooth's root. Brushing too hard can damage the gum tissue, which can lead to gum recession. Over time, the repeated trauma to the gums can cause the gums to pull back, exposing more of the tooth's surface. This can make the teeth more sensitive and vulnerable to decay and other dental problems.

To prevent gingival recession, it's important to brush gently with a soft-bristled toothbrush. You should also use proper brushing techniques, such as using circular or back-and-forth motions and holding the brush at a 45-degree angle to the teeth. Additionally, it's important to see your dentist regularly for checkups and cleanings to maintain healthy gums and prevent gum disease.

9. Are there any home remedies for gingival recession?

Gingival recession occurs when the gums pull back from the teeth, exposing the root of the tooth. While there are some home remedies that may help reduce the symptoms of gingival recession, it is important to consult with a dentist or periodontist for proper diagnosis and treatment. Here are some home remedies that may help:

Saltwater Rinse: Mix 1/2 teaspoon of salt with a cup of warm water and swish the solution around your mouth for 30 seconds before spitting it out. This can help reduce inflammation and kill bacteria in the mouth.

Oil Pulling: Swishing a tablespoon of coconut oil or sesame oil in your mouth for 15-20 minutes can help reduce inflammation and improve oral health.

Aloe Vera: Apply aloe vera gel directly to the affected area. Aloe vera has anti-inflammatory properties that can help reduce swelling and promote healing.

Green Tea: Drink green tea, which contains catechins that may help reduce inflammation and improve gum health.

Vitamin C: Increase your intake of vitamin C-rich foods, such as oranges, strawberries, kiwi, and bell peppers. Vitamin C can help promote gum health and reduce inflammation.

Remember that these home remedies may provide temporary relief, but they are not a substitute for professional dental care. If you are experiencing gingival recession, it is important to see a dentist or periodontist for proper diagnosis and treatment.

10. How often should I visit the dentist to check for gingival recession?

The frequency of dental checkups for gingival recession depends on several factors, including your age, dental history, oral hygiene practices, and overall health. However, as a general guideline, most people should visit the dentist at least twice a year for routine dental checkups and cleanings.

During these checkups, your dentist or dental hygienist will examine your teeth, gums, and mouth for signs of any dental problems, including gingival recession. They may use a special tool to measure the depth of the pockets between your teeth and gums to determine if there is any recession or gum disease.

If you have a history of gum disease, poor oral hygiene habits, or certain medical conditions that can increase your risk for gum disease, your dentist may recommend more frequent visits to monitor your condition and prevent further damage to your gums.

It's essential to follow your dentist's recommendations for dental checkups and cleanings and to practice good oral hygiene habits at home, such as brushing twice a day, flossing daily, and using an antiseptic mouthwash to help prevent gum disease and other dental problems.

D. *Periodontal Diseases*

1. What is periodontal disease?

Periodontal disease, also known as gum disease, is a chronic bacterial infection that affects the gums and supporting structures of the teeth, including the bone and ligaments. It is caused by the accumulation of plaque and tartar on the teeth and gums, which leads to inflammation and infection of the gums.

There are two main types of periodontal disease: gingivitis and periodontitis. Gingivitis is the early stage of gum disease and is characterized by red, swollen, and bleeding gums. If left untreated, gingivitis can progress to periodontitis, a more severe form of gum disease that can cause bone and tooth loss.

Risk factors for periodontal disease include poor oral hygiene, smoking, certain medications, genetics, and systemic diseases such as diabetes. Treatment for periodontal disease includes professional cleanings, scaling and root planing, antibiotics, and surgery in severe cases. Preventative measures such as regular dental cleanings, proper oral hygiene, and a healthy diet can help prevent periodontal disease.

2. What are the symptoms of periodontal disease?

Periodontal disease, also known as gum disease, can have different symptoms depending on its severity. Here are some common symptoms of periodontal disease:

Red, swollen, or tender gums

Bleeding gums, especially during brushing or flossing

Receding gums, which can make teeth appear longer

Loose or shifting teeth

Pus between the teeth and gums

Persistent bad breath or a bad taste in the mouth

Changes in your bite or the way your teeth fit together when you bite down

A change in the color of your gums from a healthy pink to a dusky red or purple

If you notice any of these symptoms, it is important to see your dentist or periodontist as soon as possible. Early treatment can help prevent further damage to your gums and teeth.

3. How is periodontal disease diagnosed?

Periodontal disease is usually diagnosed through a combination of patient history, clinical examination, and diagnostic tests. Here are the main steps involved in diagnosing periodontal disease:

Patient history: The dentist or periodontist will ask the patient about their dental and medical history, as well as any symptoms they may be experiencing, such as bleeding gums, bad breath, or tooth mobility.

Clinical examination: The dentist or periodontist will perform a thorough examination of the patient's mouth, including the teeth, gums, and supporting structures. They will look for signs of inflammation, such as redness, swelling, and bleeding, as well as measure the depth of the gum pockets around each tooth.

X-rays: X-rays can help the dentist or periodontist see the extent of bone loss and other signs of periodontal disease that may not be visible during the clinical exam.

Periodontal probing: This involves using a probe to measure the depth of the pockets around each tooth. Deeper pockets are an indication of more severe periodontal disease.

Other diagnostic tests: Depending on the severity of the periodontal disease, the dentist or periodontist may recommend other diagnostic tests, such as a saliva test to check for specific bacteria or a genetic test to determine if the patient is more susceptible to periodontal disease.

Based on the results of these tests, the dentist or periodontist will determine the stage and severity of the periodontal disease and develop an appropriate treatment plan.

4. What causes periodontal disease?

Periodontal disease, also known as gum disease, is caused by the accumulation of bacterial plaque on the teeth and gums. Plaque is a sticky film of bacteria that forms on the teeth and can harden into tartar if not removed through proper oral hygiene practices such as brushing and flossing.

When plaque and tartar are left on the teeth and gums, the bacteria can begin to irritate and inflame the gums, leading to gingivitis, the early stage of periodontal disease. If left untreated, gingivitis can progress into periodontitis, a more severe form of the disease that can cause damage to the bone and connective tissue that support the teeth, leading to tooth loss.

Other factors that can contribute to periodontal disease include smoking, hormonal changes (such as those that occur during pregnancy), certain medications, and genetic predisposition. Additionally, poor nutrition and a weakened immune system can also increase the risk of developing periodontal disease.

5. Can periodontal disease be prevented?

Yes, periodontal disease can be prevented through good oral hygiene practices, a healthy diet, and regular visits to the dentist.

Good oral hygiene practices include brushing your teeth twice a day with fluoride toothpaste, flossing daily, and using an antiseptic mouthwash. It is also important to clean your tongue and replace your toothbrush every three to four months.

A healthy diet that is low in sugar and high in nutrients can also help prevent periodontal disease. Avoiding sugary and starchy foods can help prevent the buildup of plaque on teeth, which can lead to gum disease.

Regular visits to the dentist can help prevent and detect early signs of periodontal disease. Your dentist can perform a thorough cleaning of your teeth and gums and provide recommendations for improving your oral health.

If you are at higher risk of developing periodontal disease, your dentist may recommend additional measures such as more frequent cleanings or the use of antibiotics or other treatments to prevent the disease from progressing.

6. How is periodontal disease treated?

Periodontal disease, also known as gum disease, is usually treated with a combination of professional dental care and good oral hygiene practices at home. The specific treatment plan will depend on the severity of the disease and may include the following steps:

Scaling and root planing: This is a deep cleaning procedure that removes plaque and tartar from above and below the gum line. The dentist or dental hygienist uses special instruments to remove these deposits and smooth the surface of the tooth root to promote healing.

Antibiotics: Antibiotics may be prescribed to eliminate any remaining bacteria and reduce inflammation. These can be prescribed in the form of a mouth rinse, topical gel, or pill.

Surgery: In more severe cases of periodontal disease, surgery may be necessary to remove damaged tissue and to regenerate bone and gum tissue. This can include procedures such as gum grafts, bone grafts, or flap surgery.

Maintenance: After treatment, it is important to maintain good oral hygiene practices at home and to schedule regular dental checkups and cleanings to prevent the disease from returning.

Overall, the best way to treat periodontal disease is to catch it early and take steps to prevent it from progressing. Good oral hygiene practices, such as brushing and flossing regularly, can go a long way in preventing gum disease.

7. Is periodontal disease contagious?

Periodontal disease, also known as gum disease, is not contagious in the same way as an infectious disease like the flu or a cold. However, certain factors can increase the risk of developing periodontal disease, and these factors can be shared between individuals, such as:

Bacteria: The bacteria that cause periodontal disease can be spread through saliva. For example, sharing utensils or kissing can transfer bacteria from one person to another.

Poor oral hygiene: Not practicing good oral hygiene, such as brushing and flossing regularly, can lead to an overgrowth of bacteria in the mouth and increase the risk of developing periodontal disease.

Smoking: Smoking is a risk factor for periodontal disease, and exposure to secondhand smoke can also increase the risk.

So while periodontal disease is not directly contagious, certain habits or behaviors that increase the risk of developing it can be shared between individuals. It's important to practice good oral hygiene and see a dentist regularly to prevent and treat periodontal disease.

8. How long does it take to treat periodontal disease?

The length of time required to treat periodontal disease can vary depending on the severity of the disease and the specific treatment plan recommended by your dentist or periodontist. In general, treatment for periodontal disease can take anywhere from a few weeks to several months.

For mild cases of periodontal disease, treatment may involve improved oral hygiene practices, such as more frequent brushing and flossing, and regular professional cleanings. This type of treatment may take several weeks to several months to see significant improvement.

For more severe cases of periodontal disease, treatment may involve scaling and root planing, which is a deep cleaning procedure that removes tartar and bacteria from below the gum line. This procedure may be performed over several appointments and may take several months to complete.

In some cases, surgery may be necessary to treat advanced periodontal disease. This may involve procedures such as flap surgery, bone and tissue grafts, or guided tissue regeneration. Recovery from these procedures may take several weeks to several months, depending on the complexity of the surgery.

It is important to work closely with your dental professional to develop an individualized treatment plan that addresses the specific needs of your periodontal disease and to follow through with recommended follow-up care and maintenance.

9. What is the difference between gingivitis and periodontitis?

Gingivitis and periodontitis are both types of gum disease, but they differ in their severity and the tissues they affect.

Gingivitis is a milder form of gum disease that is caused by bacterial plaque buildup on the teeth and gums. It typically results in red, swollen, and bleeding gums. Gingivitis does not involve any loss of bone or tissue that supports the teeth, and it is reversible with good oral hygiene practices, such as brushing and flossing regularly and getting regular dental checkups and cleanings.

Periodontitis, on the other hand, is a more advanced form of gum disease that can result in permanent damage to the gums and bones that support the teeth. If left untreated, it can lead to tooth loss. Periodontitis occurs when gingivitis is left untreated and the bacterial infection spreads below the gum line, causing inflammation and destruction of the gum tissue and bone. Symptoms of periodontitis include receding gums, loose teeth, and a change in the way the teeth fit together when biting.

In summary, gingivitis is a reversible inflammation of the gums caused by bacterial plaque buildup, while periodontitis is a more advanced form of gum disease that involves irreversible damage to the gum tissue and bone that support the teeth.

10. What can happen if periodontal disease is left untreated?

Periodontal disease, also known as gum disease, is a chronic bacterial infection that affects the gum tissues and bone that support the teeth. If left untreated, it can lead to several complications, including:

Tooth loss: As the disease progresses, the bacteria destroy the supporting bone and gum tissues, which can cause the teeth to become loose and eventually fall out.

Gum recession: The gums can pull away from the teeth, creating gaps or pockets between the teeth and gums. These pockets can trap bacteria, further exacerbating the infection.

Halitosis: Chronic bad breath can be a result of untreated periodontal disease, as the bacteria in the mouth produce volatile sulfur compounds that cause unpleasant odors.

Systemic health problems: Recent research has linked periodontal disease to an increased risk of several systemic health problems, such as heart disease, stroke, and diabetes.

Abscesses: Untreated periodontal disease can cause abscesses or pus-filled pockets to form in the gums or teeth, which can be painful and require emergency treatment.

Difficulty eating and speaking: As the teeth become loose and the gums recede, it can become difficult to chew or speak properly, leading to further complications and social anxiety.

It is important to seek treatment for periodontal disease as early as possible to prevent these complications and maintain good oral and overall health.

E.  Peri-implant Diseases

1. What are peri-implant diseases?

Peri-implant diseases are a group of inflammatory conditions that affect the tissues surrounding dental implants. These diseases are similar to periodontal diseases that affect natural teeth and are characterized by inflammation of the soft and hard tissues surrounding the implant.

The two main types of peri-implant diseases are peri-implant mucositis and peri-implantitis.

Peri-implant mucositis is a reversible inflammatory condition that affects the soft tissue around the implant. It is caused by bacterial plaque accumulation on the implant surface and is characterized by redness, swelling, bleeding, and tenderness of the gums.

Peri-implantitis, on the other hand, is a more severe form of peri-implant disease that affects both the soft and hard tissues surrounding the implant. It is caused by the chronic presence of bacterial plaque and is characterized by progressive bone loss around the implant, which can lead to implant failure.

Regular dental check-ups and good oral hygiene practices, such as daily brushing and flossing, can help prevent peri-implant diseases.

2. What are the symptoms of peri-implant diseases?

Peri-implant diseases are conditions that affect the soft and hard tissues surrounding dental implants. There are two main types of peri-implant diseases: peri-implant mucositis and peri-implantitis.

Peri-implant mucositis is a reversible inflammation of the soft tissues around the implant, whereas peri-implantitis is a more serious condition that involves the loss of supporting bone around the implant.

Symptoms of peri-implant mucositis may include:

Redness and swelling of the gums around the implant

Bleeding of the gums during brushing or flossing

Sensitivity or pain around the implant

Presence of pus or discharge around the implant

Foul taste or odor around the implant

Symptoms of peri-implantitis may include:

All the symptoms of peri-implant mucositis

Deepening of the gum pocket around the implant

Recession of the gums around the implant

Loose implant

Bone loss around the implant

Changes in the bite or discomfort when biting down

It is important to see a dental professional if you experience any of these symptoms, as early detection and treatment of peri-implant diseases can prevent further damage and help save the implant.

3. How are peri-implant diseases diagnosed?

Peri-implant diseases are diagnosed through a combination of clinical examination and radiographic evaluation. The diagnostic process typically involves the following steps:

Visual examination: The dentist will visually inspect the implant and surrounding tissues for signs of inflammation, swelling, redness, or bleeding.

Probing: The dentist will use a periodontal probe to measure the depth of the pocket around the implant. This helps to determine the degree of attachment loss and whether there is any bone loss.

Radiographs: X-rays can be used to detect bone loss around the implant. Radiographs can also reveal any implant-related complications such as implant fracture, implant mobility, or bone loss.

Other diagnostic tests: In some cases, other tests may be performed to help diagnose peri-implant diseases. For example, a microbial analysis may be performed to identify the specific bacteria present in the pockets around the implant.

The diagnosis of peri-implant diseases can be challenging, and it is important to seek the advice of a dental professional who has experience in implant dentistry.

4. What causes peri-implant diseases?

Peri-implant diseases are caused by the inflammation and infection of the tissues surrounding dental implants. The primary cause of peri-implant diseases is bacterial plaque, which forms on the surface of the implant and surrounding tissues. If the plaque is not removed through regular brushing and flossing, it can cause inflammation and infection of the gums, leading to peri-implant mucositis.

If left untreated, peri-implant mucositis can progress to peri-implantitis, which is a more severe form of the disease. In peri-implantitis, the inflammation and infection extend to the bone that supports the implant, leading to bone loss and potentially implant failure.

Other risk factors for peri-implant diseases include smoking, poor oral hygiene, certain systemic diseases (such as diabetes), and a history of periodontal disease. It is important for patients with dental implants to practice good oral hygiene and attend regular check-ups with their dentist to detect and treat peri-implant diseases early.

5. Can peri-implant diseases be prevented?

Yes, peri-implant diseases can be prevented with proper oral hygiene and regular dental check-ups. The following are some tips to prevent peri-implant diseases:

Brush and floss regularly: Regular brushing and flossing are essential to remove plaque and bacteria from your teeth and implants.

Use an antimicrobial mouthwash: Using an antimicrobial mouthwash can help to reduce the amount of bacteria in your mouth.

Visit your dentist regularly: Regular dental check-ups are important to monitor the health of your implants and detect any potential problems early on.

Avoid smoking: Smoking can increase the risk of peri-implant diseases, so it is best to avoid smoking altogether.

Maintain a healthy diet: A healthy diet can help to support overall oral health and reduce the risk of peri-implant diseases.

By following these tips, you can help to prevent peri-implant diseases and ensure the long-term success of your dental implants.

6. How are peri-implant diseases treated?

Peri-implant diseases are conditions that affect the soft and hard tissues surrounding dental implants, and can lead to implant failure if left untreated. Treatment for peri-implant diseases depends on the severity of the condition and may include:

Non-surgical therapy: This involves cleaning the implant and surrounding tissues using special instruments to remove bacteria and debris from the area. The goal of non-surgical therapy is to eliminate the source of the infection and promote healing.

Surgical therapy: If non-surgical therapy is not successful, surgical intervention may be necessary. This can involve removing the implant, cleaning the area, and replacing the implant.

Antibiotics: In some cases, antibiotics may be prescribed to help fight the infection and promote healing.

Maintenance therapy: After treatment, it is important to maintain good oral hygiene and schedule regular check-ups with the dentist to prevent further infection and monitor the health of the implant.

It is important to seek treatment for peri-implant diseases as soon as possible to prevent further damage to the implant and surrounding tissues.

7. Can I still have dental implants if I have a history of periodontal disease?

It is possible to have dental implants if you have a history of periodontal disease, but it will depend on the severity of the disease and the amount of damage it has caused to your teeth, gums, and bones.

Before undergoing implant surgery, your dentist or periodontist will thoroughly evaluate your dental and periodontal health to determine if you are a good candidate for the procedure. This may involve taking X-rays or 3D scans of your teeth and jawbone to assess the amount of bone density and the shape of the jawbone.

If you have a history of periodontal disease, it's important to make sure that any active infection or inflammation is under control before the implant surgery. This may involve treating any gum disease, repairing damaged teeth, or removing any teeth that are beyond repair.

In some cases, your dentist may recommend a bone graft or sinus lift procedure to improve the bone density in the implant site before the surgery. This can help ensure that the implant has a solid foundation to integrate with the jawbone and prevent complications down the line.

Overall, the decision to undergo implant surgery will depend on the unique circumstances of your dental health. It's important to have an open and honest conversation with your dental professional to determine the best course of action for your specific needs.

8. Is there a difference between peri-implant mucositis and peri-implantitis?

Yes, there is a difference between peri-implant mucositis and peri-implantitis.

Peri-implant mucositis is a reversible inflammation of the soft tissues surrounding a dental implant, without any signs of bone loss. It is characterized by redness, swelling, bleeding, and tenderness around the implant. If left untreated, peri-implant mucositis can progress to peri-implantitis.

Peri-implantitis, on the other hand, is a more severe and irreversible condition that affects both the soft tissues and the bone surrounding a dental implant. It is characterized by inflammation, bleeding, and bone loss around the implant. If left untreated, peri-implantitis can lead to implant failure.

In summary, peri-implant mucositis is a precursor to peri-implantitis and can be treated and reversed if caught early. Peri-implantitis, on the other hand, is a more advanced and destructive condition that requires more aggressive treatment and can lead to implant failure if left untreated.

9. How often should I have my dental implants checked for peri-implant diseases?

It is important to have your dental implants checked regularly to prevent and detect peri-implant diseases. The frequency of these check-ups may vary depending on your individual circumstances, such as the health of your gums, the quality of your oral hygiene routine, and any additional risk factors.

In general, most dental professionals recommend that patients with dental implants should have a comprehensive dental exam, including a check for peri-implant diseases, at least once every six months. During these check-ups, your dentist or dental hygienist will evaluate the health of your gums, check for any signs of inflammation or infection around your dental implants, and assess the stability of the implants themselves.

If you have a history of gum disease, smoke, or other risk factors, your dental professional may recommend more frequent check-ups, such as every three to four months. It is important to follow your dentist's recommended check-up schedule to ensure the long-term success of your dental implants and overall oral health.

10. How can I maintain good oral hygiene to prevent peri-implant diseases?

Peri-implant diseases, such as peri-implant mucositis and peri-implantitis, are common complications that can occur after dental implant surgery. Maintaining good oral hygiene is crucial to prevent these diseases and maintain the longevity of your dental implants. Here are some tips on how to maintain good oral hygiene and prevent peri-implant diseases:

Brush twice a day: Brush your teeth at least twice a day with a soft-bristled toothbrush. Make sure to brush gently around the implant area.

Floss regularly: Floss at least once a day to remove plaque and food particles from between your teeth and around the implant.

Use an antibacterial mouthwash: Use an antibacterial mouthwash to reduce the number of bacteria in your mouth.

Quit smoking: Smoking can impair the healing process after dental implant surgery and increase the risk of peri-implant diseases.

Visit your dentist regularly: Visit your dentist for regular check-ups and cleanings. Your dentist can detect and treat any potential issues early on.

Follow a healthy diet: A balanced diet that is rich in nutrients can help promote oral health and prevent peri-implant diseases.

Remember, prevention is always better than cure. By following these tips, you can maintain good oral hygiene and prevent peri-implant diseases.

F. *Dental Implants*

1. What are dental implants?

Dental implants are artificial tooth roots that are surgically placed into the jawbone to support replacement teeth or bridges. They are made of biocompatible materials such as titanium or ceramic and are designed to fuse with the jawbone over time, which makes them a durable and long-lasting solution for missing teeth. Once the implants have fused with the bone, abutments (connector posts) are attached to the implants, which hold the replacement teeth in place. Dental implants can be used to replace one or more missing teeth and can also be used to support a complete set of dentures. They offer many benefits over other tooth replacement options, including improved speech, better eating ability, and a more natural appearance.

2. How long do dental implants last?

Dental implants are designed to be a long-term solution for missing teeth, and with proper care, they can last for many years. The lifespan of dental implants can vary depending on several factors, including the patient's overall oral health, the quality of the implant, and the amount of wear and tear the implant undergoes.

Studies have shown that dental implants have a success rate of over 95% after five years, and many patients can expect their implants to last for 25 years or more with proper care. However, it's important to note that some implants may fail earlier due to factors such as poor oral hygiene, smoking, or underlying health conditions.

To maximize the lifespan of your dental implants, it's important to follow good oral hygiene practices, including regular brushing and flossing, as well as visiting your dentist for regular checkups and cleanings. It's also important to avoid habits that can damage your implants, such as chewing on hard objects or using your teeth to open packaging.

3. Are dental implants painful?

Dental implant placement is generally not considered a painful procedure, as it is typically performed under local anesthesia to numb the area being treated. During the procedure, you may feel some pressure or vibrations, but you should not feel any pain. After the procedure, it is normal to experience some discomfort, swelling, and bruising in the treated area. This can be managed with over-the-counter pain relievers or prescription medications, as well as ice packs and rest. Your dentist or oral surgeon will provide you with instructions on how to manage any discomfort you may experience. In general, most patients find that any discomfort associated with dental implant placement is manageable and subsides within a few days.

4. What is the success rate of dental implants?

The success rate of dental implants can vary depending on various factors such as the patient's overall health, the location of the implant, the quality and quantity of the patient's bone, and the skill of the implant dentist.

Generally, the success rate of dental implants is high, with reported success rates ranging from 90% to 98%. However, it's important to note that individual cases may vary, and some patients may experience complications that affect the success of their dental implant.

Factors such as proper oral hygiene, regular dental check-ups, and lifestyle choices such as smoking can also affect the long-term success of dental implants. It's important for patients to work closely with their dental professionals to ensure that their implants are properly maintained and cared for to maximize their success and longevity.

5. Can anyone get dental implants?

Not everyone is a good candidate for dental implants, but most people with missing teeth can get them. In general, dental implants are a good option for people who:

Have good oral health: Candidates for dental implants should have healthy gums and enough jawbones to support the implant.

Are non-smokers: Smoking can reduce the success rate of dental implant surgery.

Are committed to good oral hygiene: Proper oral hygiene is essential for the long-term success of dental implants.

Have realistic expectations: Candidates for dental implants should understand that the procedure may take several months and require multiple appointments.

Have no uncontrolled chronic diseases: Chronic diseases like uncontrolled diabetes and autoimmune diseases can affect the success rate of dental implant surgery.

Are willing to undergo surgery: Dental implant placement requires surgery, so candidates should be willing and able to undergo the procedure.

It's important to note that each person's situation is unique, and your dentist or oral surgeon will be able to determine whether you are a good candidate for dental implants after a thorough examination and evaluation.

6. How much do dental implants cost?

The cost of dental implants can vary widely depending on various factors such as the location, the complexity of the case, the type of implant used, the dentist or oral surgeon's experience, and the need for additional procedures such as bone grafting or sinus lifts.

Generally, the cost of a single dental implant can range from $1,000 to $4,000 or more, with the total cost of a full set of implants for the upper or lower jaw can cost anywhere from $20,000 to $50,000 or more.

It is important to keep in mind that while dental implants may seem expensive, they are a long-term investment in your oral health and can provide benefits such as improved appearance, speech, and chewing function, as well as prevent further dental issues in the future. It is always best to consult with a dental professional for an accurate estimate of the cost based on your specific case.

7. How long does the dental implant process take?

The dental implant process typically takes several months to complete, as it involves multiple stages and requires time for healing and integration of the implant with the jawbone. The process usually consists of the following steps:

Consultation and treatment planning: Your dentist will evaluate your oral health and discuss your treatment goals to determine if dental implants are the best option for you.

Implant placement: During this stage, the dental implant is surgically placed into the jawbone. This procedure typically takes 1-2 hours and may require local anesthesia or sedation.

Healing and osseointegration: After the implant is placed, it needs time to heal and integrate with the jawbone. This process, called osseointegration, can take several months, during which you may be given a temporary restoration.

Abutment placement: Once the implant has fully fused with the jawbone, an abutment is attached to the implant. The abutment connects the implant to the replacement tooth.

Restoration placement: The final step is the placement of the permanent restoration, such as a crown, bridge, or denture, on top of the abutment. This process usually requires two appointments.

The entire dental implant process can take anywhere from several months to over a year, depending on the individual case and the complexity of the treatment. Your dentist will provide you with a more accurate timeline based on your specific needs and circumstances.

8. Are dental implants safe?

Dental implants are generally considered safe and are one of the most effective long-term solutions for replacing missing teeth. The success rate of dental implant procedures is high, with reported success rates of up to 98 percent. However, like any surgical procedure, there are risks involved.

Some potential risks and complications associated with dental implants include infection, nerve damage, implant failure, and bone loss. Additionally, patients with certain medical conditions or who smoke may be at higher risk for complications.

It is important to discuss the risks and benefits of dental implants with your dentist or oral surgeon and to carefully follow post-operative care instructions to minimize the risk of complications. With proper planning, placement, and care, dental implants can be a safe and successful solution for restoring a healthy, functional, and aesthetically pleasing smile.

9. What are the benefits of dental implants?

Dental implants are a popular and effective way to replace missing teeth. Here are some of the benefits of dental implants:

Improved oral health: Dental implants help maintain the natural structure of the jawbone, which can prevent further bone loss and gum recession.

Better function: Implants function like natural teeth, allowing you to eat and speak with confidence.

Durability: With proper care, dental implants can last a lifetime.

Improved appearance: Implants are designed to look and feel like natural teeth, improving your smile and facial structure.

Convenience: Unlike dentures, which require removal for cleaning and can slip out of place, dental implants are permanently affixed to your mouth.

Comfort: Because they are anchored directly into your jawbone, dental implants are more comfortable than dentures and don't cause sore spots or irritation.

Improved self-esteem: Dental implants can improve your confidence by restoring your smile and allowing you to eat and speak without worry.

Overall, dental implants are a great option for people who want to restore their missing teeth and improve their overall oral health and quality of life.

10. How do I care for my dental implants?

Caring for dental implants is very important to ensure their longevity and maintain good oral health. Here are some tips for caring for your dental implants:

Brush and floss regularly: Brush your teeth twice a day and floss daily to keep your implants and surrounding teeth clean. Use a soft-bristled toothbrush and non-abrasive toothpaste to avoid damaging the implant or gum tissue.

Use a water flosser: A water flosser can be very helpful in cleaning around dental implants. It uses a stream of water to remove food particles and bacteria from hard-to-reach areas.

Visit your dentist regularly: Regular dental checkups and cleanings are important to maintain the health of your implants and detect any issues early on.

Avoid hard foods: Hard foods can put stress on your dental implants and may cause them to crack or break. Avoid chewing on ice, hard candies, or other hard objects.

Quit smoking: Smoking can increase the risk of implant failure and other oral health problems. If you smoke, quitting is one of the best things you can do for your oral and overall health.

Following these tips can help you maintain healthy dental implants and enjoy a beautiful, functional smile for years to come.

G. Periodontal Surgeries

1. What is periodontal surgery?

Periodontal surgery is a type of dental surgery that is performed to treat conditions that affect the gums and other structures that support the teeth. It is typically used to treat advanced cases of gum disease, also known as periodontitis, which can lead to the loss of teeth if left untreated.

During periodontal surgery, the dentist or periodontist (a specialist in the treatment of gum disease) will remove the diseased tissue and bacteria from around the affected tooth or teeth. They may also reshape the gums and other structures in order to promote healing and prevent further damage to the teeth.

There are several different types of periodontal surgery, including flap surgery, gingivectomy, and crown lengthening, among others. The specific type of surgery used will depend on the individual case and the severity of the gum disease. In some cases, periodontal surgery may be combined with other treatments, such as antibiotics or scaling and root planing, to achieve the best possible results.

2. Why do I need periodontal surgery?

Periodontal surgery is typically recommended when there is significant damage to the gums, periodontal ligament, or bone surrounding the teeth due to periodontal disease.

Periodontal disease is a chronic bacterial infection that affects the gums and bone supporting the teeth. In its early stages, periodontal disease may cause inflammation and bleeding of the gums, but as it progresses, it can cause pockets to form between the teeth and gums, leading to bone loss, loose teeth, and ultimately tooth loss.

Periodontal surgery may be necessary to address these issues and prevent further damage to the teeth and gums. The type of periodontal surgery recommended will depend on the severity of the disease and the extent of the damage. Some common types of periodontal surgery include gum grafts, pocket reduction surgery, and bone grafts.

It's important to address periodontal disease as early as possible to prevent the need for more invasive treatments like surgery. Regular dental check-ups and good oral hygiene can help prevent and detect periodontal disease in its early stages. If you have concerns about the health of your gums and teeth, it's important to schedule an appointment with a dental professional to discuss your options for treatment.

3. What types of periodontal surgeries are available?

Periodontal surgery refers to a range of surgical procedures performed on the gums and other tissues surrounding the teeth to treat gum disease and other conditions. Some of the most common types of periodontal surgery include:

Gingivectomy: This procedure involves removing excess gum tissue that has overgrown and is covering the teeth. It is typically performed to treat periodontal disease or to improve the appearance of the gums.

Flap surgery: This procedure involves lifting the gums to clean and remove tartar and bacteria from the roots of the teeth. The gums are then sutured back into place.

Crown lengthening: This procedure involves removing gum tissue to expose more of the tooth's surface. It is typically performed to improve the appearance of the teeth, to make them appear longer, or to prepare for a dental restoration such as a crown.

Periodontal regeneration: This procedure involves regenerating the bone and tissue that support the teeth. It is typically used to treat advanced cases of gum disease.

Soft tissue grafts: This procedure involves taking tissue from another part of the body or using synthetic material to replace gum tissue that has been lost due to gum disease or trauma.

Bone grafts: This procedure involves taking bone from another part of the body or using synthetic material to replace bone that has been lost due to gum disease or trauma.

These are just a few examples of the types of periodontal surgery available. The specific type of surgery recommended for you will depend on the extent of your gum disease or other condition and your individual needs. It is important to consult with a periodontist or oral surgeon to determine the best treatment plan for you.

4. Is periodontal surgery painful?

Periodontal surgery can cause discomfort during and after the procedure. However, the level of pain experienced varies from person to person and depends on several factors such as the type of procedure, the severity of the periodontal disease, and the individual's pain tolerance.

During the procedure, your periodontist will use local anesthesia to numb the area, so you should not feel any pain. However, you may feel pressure or a sensation of tugging as the gum tissue is being manipulated.

After the procedure, you may experience some pain, swelling, and bleeding. Your periodontist will prescribe pain medication to help manage the pain, and you may also need to take over-the-counter pain relievers. You may also need to follow specific post-operative instructions, such as avoiding certain foods and activities, to aid the healing process and reduce discomfort.

Overall, while periodontal surgery can cause discomfort, your periodontist will work to minimize your pain and discomfort during and after the procedure.

5. How long does periodontal surgery take?

The duration of periodontal surgery can vary depending on the extent and complexity of the procedure. It may range from a short 30-minute procedure to several hours for more involved procedures.

For example, a gum graft procedure to address gum recession in a single tooth may take around 30 minutes to an hour. On the other hand, more complex procedures such as bone grafting or periodontal flap surgery may take several hours to complete.

The dentist or periodontist performing the surgery will be able to give you a more accurate estimate of how long the procedure is likely to take based on your individual case.

6. What is the recovery time after periodontal surgery?

The recovery time after periodontal surgery can vary depending on the type and extent of the surgery performed, as well as individual factors such as overall health and the ability to follow post-operative instructions.

In general, it can take anywhere from a few days to a few weeks to recover fully after periodontal surgery. During this time, patients may experience some discomfort, swelling, and bleeding in the treated area.

To speed up the healing process and minimize discomfort, patients should follow all post-operative instructions provided by their periodontist or oral surgeon. This may include avoiding certain foods and drinks, taking prescribed medications, and practicing good oral hygiene habits such as brushing and flossing gently.

It's important to attend follow-up appointments with your periodontist or oral surgeon to monitor your healing progress and ensure that any potential complications are identified and addressed promptly.

7. Will I need to take time off work or school after periodontal surgery?

It is likely that you will need to take some time off work or school after periodontal surgery. The exact amount of time will depend on the type of surgery you have and how quickly you heal.

Generally, it is recommended that you take at least one day off after periodontal surgery to rest and recover. Depending on the extent of the surgery, you may need to take more time off to allow your gums to heal properly. Your dentist or periodontist will provide you with specific instructions and guidelines for post-operative care, including how long you should avoid certain activities and what types of foods you should eat.

It's important to follow your dentist's or periodontist's instructions carefully to ensure proper healing and to minimize the risk of complications. If you have concerns about taking time off work or school, you may want to discuss this with your dentist or periodontist in advance so that you can plan accordingly.

8. How do I prepare for periodontal surgery?

If you have periodontal disease and need periodontal surgery, there are several steps you can take to prepare for the procedure:

Talk to your dentist or periodontist: They can provide you with detailed instructions on how to prepare for the surgery, including any dietary or medication restrictions.

Inform your healthcare provider: Be sure to inform your healthcare provider of any medications you are taking, including over-the-counter drugs, herbal supplements, and vitamins, as these may affect your surgery or anesthesia.

Follow a healthy diet: Eating a healthy, balanced diet can help your body prepare for surgery and promote healing afterward. Make sure to eat plenty of fruits, vegetables, lean protein, and whole grains.

Quit smoking: Smoking can interfere with the healing process and increase the risk of complications during and after surgery. If you smoke, it is recommended to quit at least two weeks before your surgery.

Practice good oral hygiene: Brush and floss regularly to keep your teeth and gums clean and healthy. Your dentist or periodontist may also recommend using an antibacterial mouthwash to help reduce the number of bacteria in your mouth.

Arrange for transportation: Depending on the type of anesthesia used during your surgery, you may not be able to drive yourself home afterward. Arrange for someone to drive you to and from the appointment.

Take it easy: Plan to take it easy for a few days after your surgery to allow your body time to heal. Avoid strenuous activities or anything that may cause stress to your gums or teeth.

Remember to follow all the instructions provided by your dentist or periodontist to ensure the best possible outcome for your surgery.

9. What can I eat after periodontal surgery?

After periodontal surgery, it is important to follow your dentist's or periodontist's instructions regarding what to eat and drink. Here are some general tips:

Stick to soft and easy-to-chew foods for the first few days after surgery. Some good options include mashed potatoes, smoothies, soups, and cooked vegetables.

Avoid spicy and acidic foods, which can irritate the gums and cause discomfort.

Avoid hard, crunchy, and chewy foods, such as chips, nuts, and popcorn, which can put pressure on the gums and interfere with healing.

Avoid alcohol and tobacco products, as they can slow down the healing process.

Drink plenty of water to stay hydrated and promote healing.

You may also be advised to avoid hot or cold foods and drinks, depending on the type of surgery you had.

Always follow your dental professional's specific instructions, and contact them if you have any questions or concerns.

10. How do I take care of my mouth after periodontal surgery?

Taking care of your mouth after periodontal surgery is essential for successful healing and to prevent complications. Here are some tips to follow:

Follow your dentist's instructions: Your dentist will provide you with specific instructions on how to care for your mouth after the surgery. Follow them carefully, and if you have any questions or concerns, ask your dentist or dental hygienist.

Manage pain and swelling: You may experience pain, swelling, or discomfort after the surgery. You can manage the pain by taking over-the-counter pain relievers, applying ice packs to the affected area, and avoiding hot or spicy foods.

Take care of your oral hygiene: Brush your teeth gently and carefully twice a day using a soft-bristled toothbrush. Avoid the surgical area for the first 24 hours, and then begin to clean it gently with a toothbrush and warm saltwater rinse.

Avoid smoking: Smoking can delay healing and increase the risk of complications. It's best to avoid smoking or using any tobacco products for at least 48 hours after the surgery.

Eat a soft and nutritious diet: Stick to soft and easily chewable foods like soups, mashed potatoes, and smoothies for the first few days after the surgery. Avoid hard, crunchy, or sticky foods that can irritate the surgical site.

Attend follow-up appointments: It's essential to attend all follow-up appointments with your dentist to monitor your healing progress and address any issues that may arise.

Remember that proper care of your mouth after periodontal surgery can promote healing, prevent complications, and improve the long-term health of your gums and teeth.
